# A Novel Sorbitol-Based Flow Cytometry Buffer Is Effective for Genome Size Estimation across a Cypriot Grapevine Collection

**DOI:** 10.3390/plants13050733

**Published:** 2024-03-05

**Authors:** Kyriakos Michael, Constantina Andreou, Anastasia Markou, Michalakis Christoforou, Nikolaos Nikoloudakis

**Affiliations:** Department of Agricultural Science, Biotechnology and Food Science, Cyprus University of Technology, Limassol 3036, Cyprus; km.michael@edu.cut.ac.cy (K.M.); cos.andreou@edu.cut.ac.cy (C.A.); ag.markou@edu.cut.ac.cy (A.M.); m.christoforou@cut.ac.cy (M.C.)

**Keywords:** C-value, DNA content, sorbitol, tannins, *Vitis vinifera*

## Abstract

Flow cytometry (FCM) is a widely used technique to study genome size (C-value), but recalcitrant metabolites in grapevines often hinder its efficiency in grapevine research. The aim of the present study was (i) to develop a novel buffer tailormade for the nuclei isolation of grapevines and (ii) to characterize a Cypriot germplasm collection based on C-values. A local cultivar “Xinisteri” was used as a pilot test to evaluate a Sorbitol-based buffer, while sprouting, young, and fully matured leaves were examined to evaluate the developmental parameter. The novel Sorbitol buffer was shown to have a coefficient of variation (CV) of 4.06%, indicating improved properties compared to other commonly used FCM buffers [WPB (7.69%), LB01 (6.69%), and LB (7.13%), respectively]. In addition, a significant variation in genome size between genotypes was found in a comprehensive application with 24 grape varieties. Nucleic content (2C) ranged from 0.577/1C pg for the “Assyrtiko” cultivar up to 0.597/1C pg for the “Spourtiko” cultivar, revealing a 17.6/1C Mbp difference. The lowest coefficient of variation (CV) across all entries was found in the variety “Ofthalmo” (2.29%), while the highest was observed in “Pinot Noir” (3.44%). Anova analysis revealed several distinct clusters, showing that in several cases, C-values can be used as a simple method to distinguish grapevine cultivars.

## 1. Introduction

Since flow cytometry (FCM) was introduced in plant science research, it has been mainly used for the determination of nuclear DNA content [1]. The popularity of the method has been aided by the ease of sample preparation, which usually requires mechanical homogenization of plant tissue in a buffer for nuclear isolation [2]. Ideally, an FCM buffer should facilitate the isolation of intact nuclei (free of cytoplasmic debris), keep the nuclei stable in the liquid suspension, and also prevent them from aggregating so that laminar flow is maintained [3]. In addition, it should protect the nuclear DNA from hydrolysis/degradation while providing sufficient background for stoichiometric and exclusive labelling of nuclear DNA, while minimising the deleterious effects of cytosolic contents on DNA staining.

Nevertheless, the transfer of working protocols from animal/microbial samples to plants has not been straightforward due to the dissimilar biochemical nature between the different taxonomic kingdoms and the complexity of metabolites found in (mostly perennial) plants [4]. Secondary metabolites are usually systemically produced in plants and further upregulated when plants are exposed to biotic or abiotic influences [5]. Thus, plants accumulate carotenoids, anthocyanins, polyphenols, and other ROS-scavenging compounds. Such metabolites are useful as they can protect cellular integrity by scavenging oxygen or nitrogen free radicals and mitigate cell damage caused by abiotic (e.g., salinity, solar radiation, etc.) and/or biotic factors [6]. In addition, they act as a natural barrier to maintain the integrity and homeostasis of the cell/plant.

In grapevines, the typical secondary metabolites are tannins [7] and thus tannic acid, which is often a decisive factor in the production of good or poor-quality wines. Tannins are known as bitter and astringent substances. They are polyphenolic chemicals that are divided into two main types: hydrolyzable tannins and condensed tannins (proanthocyanidins) [8]. There are hundreds of different compounds, but they all have a few properties in common. Tannins bind proteins/cell walls [9], which justifies their name (this phenomenon of tannin binding comes from the Latin phrase “ad astringere”, meaning “to bind”). Recently, more than 130 phenolic constituents have been detected in grapevine leaves, most of which are classified as tannins and flavonoids, each reaching concentrations of up to 10 mg/g of tissue [7].

Nevertheless, the nature of these compounds causes problems in the downstream processes of nucleic acid manipulation and the proper binding of fluorochromes for FCM [10]. Several cytosolic components have been proposed as interfering molecules [4,11,12,13,14,15], but the conclusions were uncertain. In a pioneering work, Loureiro and colleagues described the interfering effects of tannic acid on the estimation of C-values and the cell cycle due to the formation of clumps, distorted fluorescence features, and light scattering from nuclei in suspension [10]. As grapevines are deciduous plants, the negative effects on downstream processing increase as leaves mature, and tannins/tannins build up [5]. Nevertheless, the difficulties in downstream processing of nucleic acid are mainly associated with the genotype factor [16,17].

To address such phenomena, tissues from embryos and anther cultures [18,19], in vitro plantlets [20], regenerated plants [21], fresh young leaves [22], young leaves with trichomes removed [23], incompletely expanded young leaves [24], petioles [25], dormant axillary buds [26], and newly formed shoot tips [27] have been regularly used. However, the omission of mature leaves for FCM entails some limitations, such as the impossibility of studying the cell cycle at late developmental stages, the obligatory use of in vitro techniques, or simply the fact that field sampling is literary limited to a few days when leaves are relatively young and not fully expanded. In a recent study, we found that Sorbitol-based buffers are able to neutralise recalcitrant metabolites, such as tannins, in the downstream processing of grapevine nucleic acids [28]; this shows that there is also room for improvement in the composition of buffers for nuclei isolation and FSM.

The improvement of FCM processes in grapevines also holds particular potential for Cyprus. Cyprus is an island between three continents, a hotspot of biodiversity and a centre of domestication of *Vitis* spp. species [29]. The geographical isolation due to different microclimates as well as the presence of civilization, trade, and agriculture since ancient times has contributed to the formation of numerous exclusive genotypes in the different prefectures [30,31,32,33,34]. Therefore, from an evolutionary point of view and with regard to the exploration/utilization of genetic resources, there is a scientific interest in the characterisation of the Cypriot grapevine gene pool, especially since Cyprus is a phylloxera-free zone with unexplored genotypes.

## 2. Materials and Methods

### 2.1. Plant Material

For the test of recalcitrant plant material, an in situ sampling of pest-free leaves took place at CUT’s Lofou farm in mid-September 2022 (mature leaves) and early April 2023 (young leaves). The plants were marked, and leaves from the third node onwards were sampled (Supplementary Figure S1). Freshly cut leaves were then placed between wet paper towels and stored in zippered bags at a low temperature (4 °C) until analysis (tests were conducted within two days). Taxa used as internal flow cytometry standards (*Solanum lycopersicon* cv. Stupické polní tyčkové rané (1C = 0.98 pg)); *Raphanus sativus* cv. Saxa (0.55 pg/1C) were obtained from Prof Doležel/the Centre of Plant Structural and Functional Genomics (Šlechtitelů 31, Holice, 77900 Olomouc, Czechia) and selected on the basis of genomic size proximity to *Vitis* spp. samples. *Hordeum vulgare* cv “Achna” (9.16 pg/1C) was used as a control due to the low interfering metabolite levels of cereals. For the analysis of C-values in the vine core collection (sprouting leaves), several canes per cultivar (from the previously marked vines) were pruned (January 2023), planted in pots with a commercial perlite/peat mixture, and kept in a growth chamber (20–25 °C, 16 h/8 h photoperiod) until bud brake (March 2023). 

### 2.2. Sample Preparation

In a Petri dish (placed on top of ice), a leaf area of approximately 0.5 cm^2^ per vine sample and standards were chopped together for 10 to 20 s using a sterile double-edged razor blade per sample. The tissues were immersed in one millilitre of pre-chilled buffer(s) (Table 1). Homogenates were passed through 30 μm Celltrics nylon filters (Sysmex, Lincolnshire, IL, USA) into 1.5 mL Eppendorfs and held at 8 °C for 10 min to increase/improve staining. A total of 24 grapevine cultivars were analysed (Table 2).

### 2.3. Flow Cytometry

The ability of buffers to isolate nuclei and estimate accurate C-values was assessed using a BD Accuri C6 flow cytometer (Accuri Cytometers, Inc., Ann Arbor, MI, USA), as previously described [36]. The analysis was based on light scattering and fluorescence signals generated by a 20 mW laser at 488 nm. The precision of the cytometer was determined using 8-peak Spherotech fluorescent beads, according to the manufacturer’s instructions (CFlow User Guide, Accuri). To exclude irrelevant debris from detection, two thresholds were chosen (80,000 on FSC-H and 1000 FL-2). Fluidic flow was set to slow, and data were collected to a total of 5 min/3000 nuclei. The nuclei’s regions were diagonally gated using an FL3-A/FL2-A plot, and the peaks were displayed using a count/FL2-A function. For each accession, three different measurements were performed on three consecutive days. The replicates were well reproducible, with low systematic errors. To determine the simulated peaks for C-value estimates, flow data were exported and examined with Modfit LT version 5.0 (Verity Software House, Topsham, ME, USA). The flow histograms appeared as sharp peaks with a low coefficient of variation (<5%).

The genomic content of each cultivar was determined using the following formula:

$$1C\;nuclear\;DNA\;content\;of\;sample\;(pg)=\frac{sample\;G0/G1\;mean\;FL\times 1C\;nuclear\;DNA\;content\;of\;reference\;standard}{reference\;standard\;G0/G1\;mean\;FL}$$

The mass values (pg) were converted into the number of base pairs (Mbps), as previously reported [37].

### 2.4. Statistical Analyses

C-value averages and standard deviations were calculated for each cultivar (across all replicates) and are reported in Table 2. The Shapiro–Wilk test was used to assess variance uniformity and the fit to a normal distribution. A one-way ANOVA test was used to analyse the differences between cultivars, followed by a Tukey’s honest significant difference (HSD) test. The agricolae package and Rstudio (version 1.1.463) were used for the analyses.

## 3. Results

**Figure 1:**
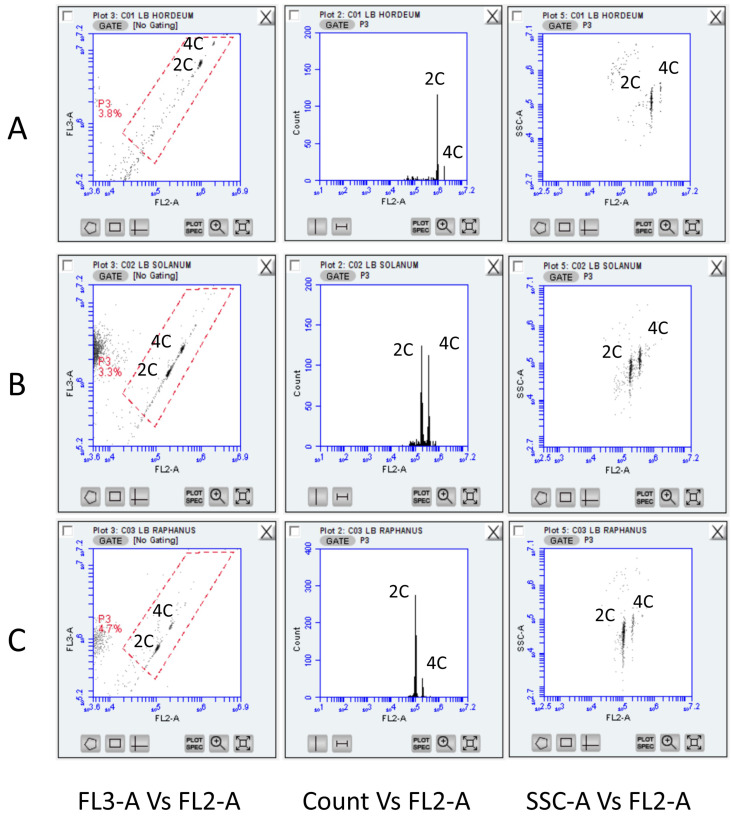
Representative FCM histograms across species used to define the gating region (P3; in red quadrilateral). The LB01 buffer was used employing (**A**) *Hordeum vulgare* cv “Achna” (5.33 pg/1C); (**B**) *Solanum lycopersicon* cv. Stupické polní tyčkové rané (0.98 pg/1C) and (**C**) *Raphanus sativus* cv. Saxa (0.55 pg/1C) leaves. Across taxa, a CV lower than 5% was established and little debris was detected within the gated area. FL2 and FL3 refer to the standard filter configuration for BD Accuri C6 (FL2: 585/40; FL3: 670/LP). 2C and 4C cell populations were easily identified across all filters and scattering plots.

Initially, tests were performed using Lysis Buffer 01, which, according to the literature, is one of the most commonly used solutions for FSM in plant species such as corn, peas, tomato, grapevine, etc. In all species (barley, radish, and tomato), a clear distinction of 2C and 4C cell populations was achieved. The plot of the FL3-A vs. FL2-A function revealed two populations and an FL2-A acquisition without axis distortion (Figure 1). In all cases, the coefficients of variation (CV) were < 5%, suggesting an excellent discriminatory power [10]. C-value standards (*Solanum lycopersicon* cv. Stupické polní tyčkové rané, *Raphanus sativus* cv. Saxa, and *Hordeum vulgare* cv “chna”) were used in order to identify 2C and 4C cell populations at an increasing diagonal scale. This allowed the selection (gating) of a free-of-debris area. Based on genomic content values of grapevine from the literature (approximately 0.6 pg/1C), 2C nuclei of grapevines should be placed between radish 2C and tomato 2C nuclei populations.

**Figure 2:**
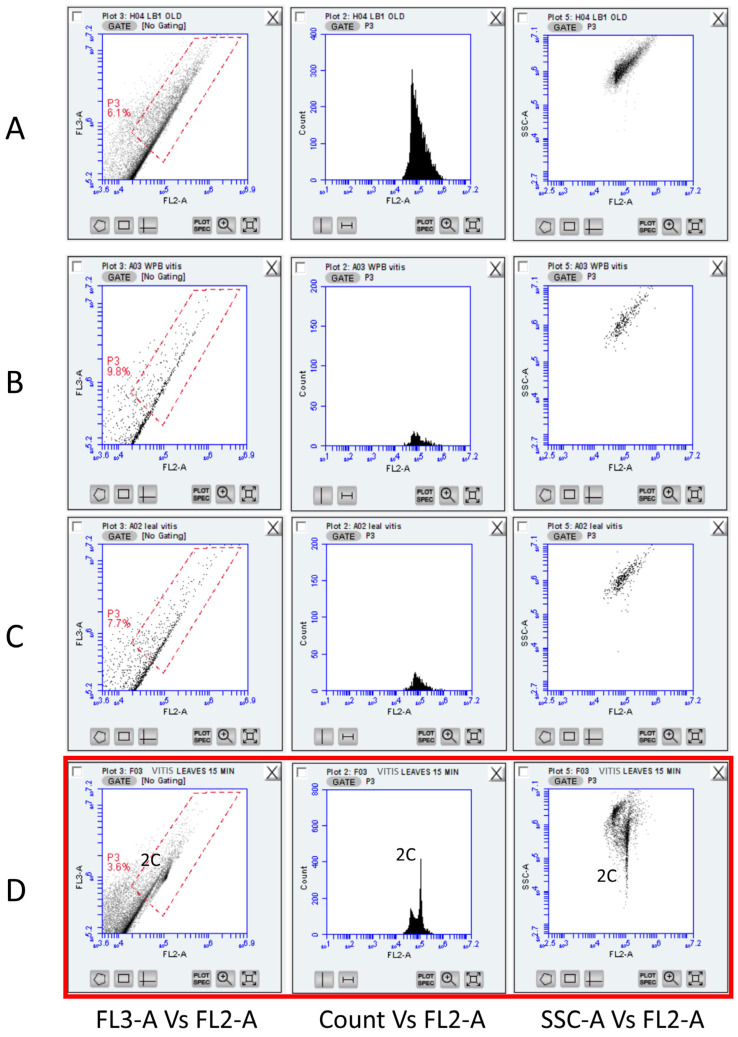
Representative FCM histograms across buffers employed to detect nuclei from mature leaves (field-collected) of *Vitis vinifera* cv “Xynisteri”. (**A**) Lysis buffer LB01; (**B**) Woody-Plant Buffer (WBP); (**C**) Leal’s buffer; and (**D**) Sorbitol-based buffer (SBB). A 2C population of nuclei was prominent only in the case of Sorbitol-based buffer (red box) and grapevine nuclei populations could be identified using a second diagonal gate (SSC-A vs. FL2-A). FL2 and FL3 refer to the standard filter configuration for BD Accuri C6 (FL2: 585/40; FL3: 670/LP).

However, in mature grape leaves (Supplementary Figure S1), nuclei populations could not be distinguished when using LB01 buffer due to interference from metabolites, while measurements of FL3-A versus FL2-A and counting versus FL2-A showed a continuous range of debris or aggregates (Figure 2A). In addition, testing mature leaves using two other typical nuclei buffers for FCM [Leal’s solution and Woody Plant Buffer (WPB)] confirmed that the high level of recalcitrant constituents in all assay modes (FL3-A vs. FL2-A, SSC-A vs. FL2-A, and Count vs. FL2-A) presented obstacles to detecting and analysing grapevine nuclei (Figure 2B,C). Next, we used the same plant material (the same ripe grapevine leaves) and the Sorbitol–glycine-based nuclei isolating buffer (Figure 2D). The use of the Sorbitol–glycine solution resulted in a significant improvement, as 2C nuclei were detectable despite a large amount of debris. 4C populations were not easily identified, probably because cell proliferation at the mature developmental stage was minimal. Nonetheless, the Sorbitol-based buffer reduced tannic acid phenomena, and events were linearly placed in the FL2-A axis (versus SSC-A). Moreover, 2C nuclei were easily detected across all modes of analyses, while phenolic oxidation was minimal compared to the other buffers. 

**Figure 3:**
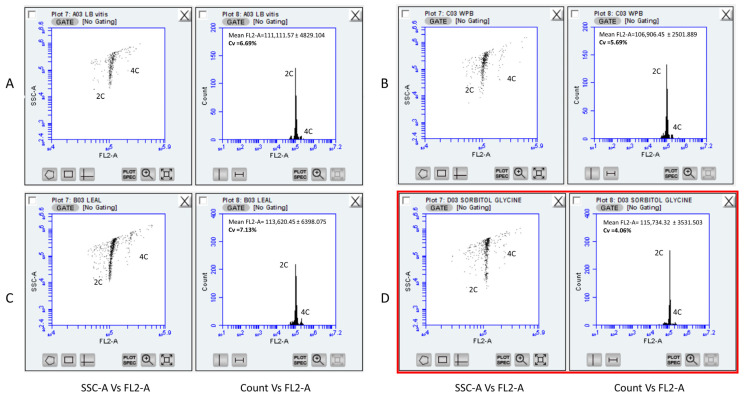
Representative gated FCM histograms across buffers used to detect nuclei from young leaves (field-collected) of *Vitis vinifera* cv “Xynisteri”. (**A**) Lysis buffer LB01; (**B**) Woody-Plant Buffer (WBP); (**C**) Leal’s buffer; and (**D**) Sorbitol-based buffer (SBB). The lowest CV (<5%) resulted from a minimum curved/distorted FL2-A axis and was noted for the Sorbitol-based buffer (red box). FL2 refers to the standard filter configuration for BD Accuri C6 (FL2: 585/40). 2C and 4C cell populations were identified across all filters and scattering plots.

To compare the Sorbitol-based Buffer with commonly used FCM buffers (LB01, Leal’s, and WPB), younger (field-collected) grapevine leaves were employed (Figure 3). Despite the presence of particles/debris in the gated region, an adequate number of nuclei required for a satisfactory analysis was obtained and analysed. The number of nuclei attained by using all four buffers ranged from 5758 ± 897/mL in LB01 to 13,291 ± 4272/mL in Leal’s buffer. The Sorbitol-based Buffer yielded 7875 ± 835 nuclei/mL that were mostly free of clumps/debris (Supplementary Figure S2). The highest FL2-A value (115,734.32 ± 3531.503) was observed for the nuclei isolated by the Sorbitol-based buffer, followed by Leal’s and LB01 buffers (113,620.45 ± 6398.075 and 111,111.57 ± 4829.104, respectively). 

Finally, the lowest CV in the G0/G1 populations was obtained from the nuclei extracted from the Sorbitol-based buffer (4.06%), which is almost 45% better than the buffer with the highest CV (Leal’s buffer; CV = 7.13%). Hence, it was established that the novel composition significantly minimised background elements and had an analogous yield compared with typical FCM buffers. Moreover, the percentage of nuclei versus the percentage of debris across the buffers was evaluated. WPB had a better (%) nuclei to (%) debris ratio (1.966 ± 0.208), followed by Leal’s (1.767 ± 0.153) and Sorbitol-based buffer (1.433 ± 0.115), while LB01 produced the largest background (1.333 ± 0.127). 

**Figure 4:**
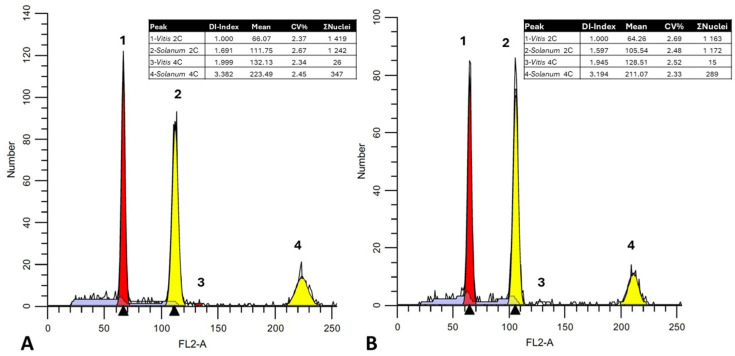
FL2-A fluorescence histograms of *V. vinifera* cv. “Assyrtiko” (**A**) and cv. “Spourtiko” (**B**) depicted as red histograms. *Solanum lycopersicon* cv. Stupické polní tyčkové rané (yellow peaks) with 2C and 4C nuclei (ModFit LT^TM^ was used for visualization). The DNA index (DI; mean channel number of sample/mean channel number of reference standard), mean channel number, coefficient of variation (%CV), and number of particles are indicated in the upper right box. Black triangles indicate the mean fluorescence value. In the current figure DI values (1.691 and 1.597) support the discrete cultivars clustering by Anova (Table 2).

The Sorbitol-based buffer was also used to estimate the genomic content across a Cypriot grapevine core collection comprising 11 Cypriot, eight French, four Greek, and one Spanish cultivar (Table 2). These grapevine plant genetic resources were found to be quite rich in genomic diversity. In total, 72 grapevines were analysed (three cultivar clones were used as a composite sample for each cultivar). Estimated C-values between grapevine taxa were calculated based on genomic proximity using the genome size of an appropriate internal reference standard (*Solanum lycopersicum* cv. Stupické polní statykové rané; 1.96 pg). In all cases, the 2C peak (FL2-A axis) of the tomato reference sample was placed within the 2C and 4C peak range of the vine sample, contributing to accurate estimates of DNA content (Figure 4; Table 2).

Particles, as shown by FL2-A histograms, were mainly clustered as two separate peaks: a lower peak matching to G2 phase nuclei (4C-value) and a single unique peak corresponding to G0/G1 phase nuclei (2C-value). Estimation of the FCM events showed that most nuclei were in the G0/G1 cell cycle. When analysing samples, high-resolution histograms with an FL2-A coefficient of variation (CV) lower than 5% were observed (Table 2). The mean CVs between biological replicates on three consecutive days (using the same batch of buffer), were also low (approximately 1.5%), indicating accurate measurements.

**Figure 5:**
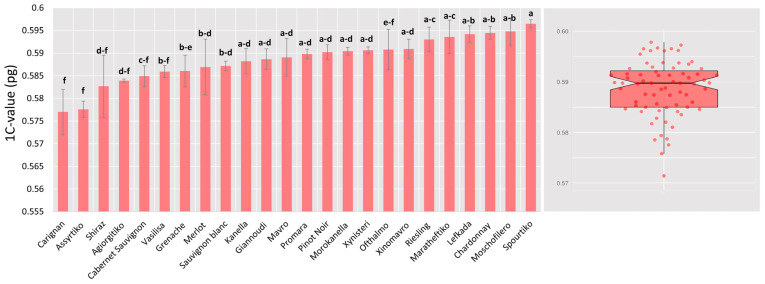
Means and distribution of 1C-values (linear scale) across grapevine cultivars (**left**). A Shapiro–Wilk test for normality supported a normal distribution hypothesis (*p* = 0.24). Cultivars having identical letters were not found significantly different at *p* = 0.05. Boxes above and below the mean line indicate quartiles (**right**).

The 1C and 2C genome size for all grapevine entries was calculated on three consecutive days. It was found that the average 1C and 2C genomic size varied significantly for the different genotypes, ranging from 0.578 pg (1C) for cv. “Assyrtiko” to 0.596 pg for cv. “Spourtiko”. This difference in DNA content corresponds to a variation of approximately 17.6 Mbp, which should be considered substantial since the grapevine genome has one of the smallest C-values of any major crop. Finally, a boxplot diagram was constructed, and it was established that C-values followed a normal distribution (Figure 5), with a median of 0.589 pg/1C and a mean of 0.589/1C. A Shapiro–Wilk test (*p* = 0.24) was used to determine the normal distribution, and equal variances were determined for post hoc analysis (HSD test). Analysis of variance (Anova) showed that DNA content variation between cultivars was typically negligible, hence taxa were assigned to similar clusters (Figure 5; Table 2). Nevertheless, there were cases where there were significant C-value disparities between varieties, and cultivars were assigned to separate clusters. As a result, C-values could not always be used as discrete traits when assigning cultivars.

## 4. Discussion

Obtaining intact nuclear suspensions is of central importance for the determination of the absolute DNA content using FCM. However, this is often complicated by the chemical components of the disrupted cell; hence, the removal of such moieties determines the quality of the nuclei [4]. Since many of the typical buffers for nucleus isolation in grapevine do not work reliably, the aim of this work was to standardise a nuclei isolation that is able to isolate intact nuclei from young and mature grapevine leaves.

Numerous recalcitrant metabolites are found in grapevines that can interfere with the released nuclei in the cytoplasmic lysate, by producing pseudo-fluorescence or coagulation. The most widespread compounds among them are polyphenols, tannins, flavonoids, and various viscous hydrophobic substances [7]. Another problem comes from the nuclei isolation protocol per se. Nuclei suspensions are released by mechanical chopping, resulting in the inclusion of Ca_2_ cations, debris of viscid membranes, as well as cell-wall components such as cellulose, hemi-cellulose, and pectins forming clumps [38]. These (mostly hydrophobic) residues cause individual nuclei to clump together (as nuclei are protected by a bilayer lipid membrane), making the samples inappropriate for genome content estimation. In addition, high concentration of tannins/tannic acids in grapevines exacerbates these errors. Tannic acids are common constituents found mainly in woody plants [10]. Greilhuber [39] first reported that tannins can cause errors in Feulgen staining by limiting the interactions between DNA and Schiff’s reagent. Moreover, it was also suggested that tannins can interact with various proteins of the nucleus. 

Such phenomena are exaggerated and correlate with the age of the leaves, as developmental processes promote the accumulation of these substances [5]. As a response, scholars mostly use young tissue, or tissue culture-produced material to minimise interference with the downstream FCM process. Still, this tendency limits the possibilities of field sampling, the timeframe of research intervals, as well as analyses focusing on development. 

In the present work, we have formulated a nuclei-isolating buffer based on past knowledge of chemical components and interactions. In the work of Grigoriou and colleagues [28], it was reported that Sorbitol binds to polyphenols and polysaccharides and thus neutralizes them before they can covalently bind to nucleic acids. The binding capacity of amino acids such as proline and glycine has also been previously confirmed by using tannin–protein interactions [40,41,42]. Additionally, the antioxidant capabilities of glycine (probably by preventing the formation of free radicals) are well documented [43]. EDTA has been used as a chelating agent for divalent cations, which acts as a co-enzyme for nucleases, in order to protect against the degradation of nuclear DNA [44]. In addition, previous studies have shown that Polyvinylpyrrolidone 40 (PVP-40), as a phenolic binder, attenuates the effect of polyphenols by forming hydrogen bonds with them, changing their conformational shape and keeping them in a reduced state [45]. Culture-tested Tween 20 was used as a non-ionic surfactant to facilitate stronger plastid lysis and to reduce the number of fluorescent particles [46]. Beta mercaptoethanol was included to establish reducing conditions, prevent the oxidation of phenolic moieties, and reduce hydrophobic interactions in combination with Polyvinylpyrrolidone [46]. Tris-buffered saline (supplemented with 90 mM NaCl) was used as an isotonic solution to keep the pH (7.5) value stable throughout manipulations. A slightly alkaline buffering capacity contributes to the hydrolysis of RNA [47], which contributes to an accurate assessment of DNA content and thus lower CVs.

Testing of the novel Sorbitol-based buffer showed an ability to produce suitable nuclei suspensions across developmental stages of grapevine leaves (from sprouting to fully mature leaves) with a relatively small amount of debris and non-specific fluorescence. Moreover, a low CV (<5%) was achieved even in cases when the identification of nuclei population in other typical FCM buffers (LB01, Leal’s buffer, WPB) was not possible. A comparison between buffers using young cv. “Xynisteri” leaves (field-collected) also showed that the novel Sorbitol-based buffer performed better in yield and quality, achieving a 4% CV, while other buffers in general produced CVs larger than 5% (Figure 3). Moreover, in the case of sprouting leaves, CV values were steadily lower than the 3% (2.688 ± 0.318) range (Table 2). In terms of nuclei percentage over the number of debris, the Sorbitol-based buffer was outperformed mainly by the WPB, but it performed better than the LB01 buffer, having a 1.433 ± 0.115 nuclei-to-debris percentage. 

Following the pilot experiments, a comprehensive test was carried out with 24 different grapevine samples (mainly of Cypriot origin). Across biological replicates (72 samples), high-resolution histograms were produced having a 2C peak coefficient of variation (<5% CV). The average coefficients of variation between biological replicates over three consecutive days (using the same buffer batch) were also low (less than 1.5%), indicating accurate measurements (Table 2). The data presented here are the first reported on the genome size of Cypriot grapevine germplasm, which is among the oldest known cultivars in the world, at five millennia [48]. 

1C-values ranged from 0.577 pg/1C for the variety “Assyrtiko” to 0.597 pg/1C for the variety “Spourtiko”, showing a 17.6 Mbp/1C difference across genotypes. Considering that the entire haploid grapevine genome has been estimated to be approximately 500 Mbp [49], this constitutes a significant difference. Furthermore, the Kew Plant DNA C-values Database (assessed in January 2024), gives a 2C mean value of 1.07 pg for grapevines. Nonetheless, this discrepancy can be attributed to the use of different genome quantification techniques, such as Feulgen densitometry, or even the use of different FCM standards regarding chicken cells [22,23]. The significance of selecting appropriate standards has been extensively discussed, and nowadays most scholars use a specific collection of standards ranging from 1.11 pg/2C (*Raphanus sativus* L. ‘Saxa’) to 34.89 pg/2C (*Allium cepa* L. ‘Alice’) as previously reported [46]. In the present study, the use of *Solanum lycopersicon* cv. Stupické polní tyčkové rané (1.96 pg/2C) must be considered the ideal standard since 2C *S. lycopersicum* populations are identified between 2C and 4C *Vitis vinifera* L. nuclei without interceptions (Figure 4A).

As reported by Gonzalez and co-workers [22], a clear distinction across berry types and skin colour was not possible. This was also the case in our grapevine collection, as berry traits and origins could not be attributed to genome size. Nonetheless, outgroup values were noted for “Carignan”, and “Assyrtiko” having a significantly smaller genome than “Moschofilero” and “Spourtiko”; indicating that FCM can be used as a screening technique across cultivars in some cases. 

## Figures and Tables

**Table 1:** Composition of nuclei isolation buffers used for grapevine analyses.

Buffer	Chemical Composition
Lysis Buffer (LB01); [35]	15 mM Tris HCl; 2 mM Na_2_EDTA; 0.5 mM Spermine tetrahydrochloride; 80 mM KCl; 20 mM NaCl; 0.1% (*v*/*v*) Triton X-100; 0.1% (*v*/*v*) β-mercaptoethanol; 50 μg/mL RNAse; 50 μg/mL Propidium Iodide; pH 7.5
Leal’s buffer; [23]	100 mM Tri-Sodium Citrate; 50 mM HEPES; 5 mM EDTA; 50 mM Glucose; 15 mM NaCl; 15 mM KCl; 1% (*w*/*v*) Polyvinylpyrrolidone (PVP-40); 1% (*v*/*v*) Tween 20; 0.1% (*v*/*v*) β-mercaptoethanol; 50 μg/mL RNAse; 50 μg/mL Propidium Iodide, pH 7.2
Woody-Plant Buffer (WBP); [3]	0.2 mM Tris HCl; 2 mM MgCl_2_; 2.5 mM EDTA; 86 mM NaCl; 10 mM Sodium sulphite; 1% (*w*/*v*) Polyvinylpyrrolidone (PVP-40); 1% (*v*/*v*) Triton X-100; 0.1% (*v*/*v*) β-mercaptoethanol; 50 μg/mL RNAse; 50 μg/mL Propidium Iodide; pH 7.5
Sorbitol-based buffer (SBB); Current-first report	100 mM Tris-HCl; 0.35 M Sorbitol; 0.05 M glycine; 5 mM EDTA; 90 mM NaCl; 1% (*w*/*v*) Polyvinylpyrrolidone (PVP-40); 0.5% (*v*/*v*) Tween 20; 0.1% (*v*/*v*) β-mercaptoethanol; 50 μg/mL RNAse; 50 μg/mL Propidium Iodide; pH 7.5

**Table 2:** Cultivar nomenclature (Vitis International Variety Catalogue VIVC; https://www.vivc.de/ accessed on 5 January 2024) and genomic attributes across the studied grapevines.

Cultivar/Prime Name	Variety Number VIVC	Country/Region of Origin	Colour of Berry Skin	1C-Value (pg)	2C-Value (pg)	2C-Mbp	CV (%)	HSD ^1^
Agiorgitiko	102	Greece	Noir	0.5839 ± 0.0026	1.1678 ± 0.0052	1142.1700	2.900 ± 0.200	d-f
Assyrtiko	726	Greece	Blanc	0.5776 ± 0.0018	1.1552 ± 0.0036	1129.7910	2.507 ± 0.203	f
Cabernet Sauvignon	1929	France	Noir	0.5849 ± 0.0023	1.1698 ± 0.0046	1144.1110	2.723 ± 0.152	c-f
Carignan	2098	France	Noir	0.577 ± 0.005	1.154 ± 0.01	1128.6550	2.453 ± 0.397	f
Chardonnay Blanc	2455	France	Blanc	0.5945 ± 0.0015	1.189 ± 0.003	1162.7610	2.463 ± 0.182	ab
Giannoudi	-	Cyprus	Noir	0.5887 ± 0.0023	1.1774 ± 0.0046	1151.4230	2.643 ± 0.145	a-d
Grenache	4461	Spain	Noir	0.586 ± 0.0035	1.172 ± 0.007	1146.3040	2.607 ± 0.136	b-e
Kanella	16,124	Cyprus	Blanc	0.5882 ± 0.0028	1.1764 ± 0.0056	1150.5570	2.417 ± 0.144	a-d
Lefkada	-	Cyprus	Noir	0.5942 ± 0.0018	1.1884 ± 0.0036	1162.2620	2.637 ± 0.543	ab
Maratheftiko	7374	Cyprus	Noir	0.5936 ± 0.0037	1.1872 ± 0.0074	1161.0500	2.497 ± 0.172	a-c
Mavro	27,628	Cyprus	Noir	0.5891 ± 0.0042	1.1782 ± 0.0084	1152.2250	2.667 ± 0.095	a-d
Merlot Noir	7657	France	Noir	0.5869 ± 0.0061	1.1738 ± 0.0122	1148.0270	2.690 ± 0.639	b-d
Morokanella	16,123	Cyprus	Noir	0.5904 ± 0.0009	1.1808 ± 0.0018	1154.8720	2.587 ± 0.341	a-d
Moschofilero	8068	Greece	Rose	0.5948 ± 0.003	1.1896 ± 0.006	1163.4160	2.607 ± 0.090	ab
Ofthalmo	8782	Cyprus	Noir	0.5908 ± 0.0045	1.1816 ± 0.009	1155.5940	2.293 ± 0.107	ef
Pinot Noir	9279	France	Noir	0.5902 ± 0.0017	1.1804 ± 0.0034	1154.4150	3.440 ± 0.104	a-d
Promara	9737	Cyprus	Blanc	0.5898 ± 0.0011	1.1796 ± 0.0022	1153.6940	3.153 ± 1.268	a-d
Riesling	3264	France	Blanc	0.593 ± 0.0027	1.186 ± 0.0054	1159.9250	2.317 ± 0.057	a-c
Sauvignon blanc	10,790	France	Blanc	0.5872 ± 0.0011	1.1744 ± 0.0022	1148.5260	2.413 ± 0.108	b-d
Shiraz	11,748	France	Noir	0.5827 ± 0.0069	1.1654 ± 0.0138	1139.7350	2.893 ± 0.180	d-f
Spourtiko	16,121	Cyprus	Blanc	0.5965 ± 0.0009	1.193 ± 0.0018	1166.7150	3.023 ± 0.785	a
Vasilisa	-	Cyprus	Blanc	0.5859 ± 0.0013	1.1718 ± 0.0026	1146.0950	2.973 ± 0.873	b-f
Xinomavro	13,284	Greece	Noir	0.5909 ± 0.0021	1.1818 ± 0.0042	1155.8570	2.940 ± 0.583	a-d
Xynisteri	704	Cyprus	Blanc	0.5906 ± 0.0008	1.1812 ± 0.0016	1155.2420	2.660 ± 0.131	a-d
Average	-	-	-	0.5886 ± 0.0026	1.1773 ± 0.0052	1151.3926	2.688 ± 0.318	-

^1^ Cultivars having identical letters were not found significantly different at *p* = 0.05.

## Data Availability

Data are contained within the article and supplementary materials.

## Supplementary Materials

The following supporting information can be downloaded at: https://www.mdpi.com/article/10.3390/plants13050733/s1, Figure S1. An example of a fully matured leaf of cv. “Xynisteri”. Accumulation of pigments due to UV exposure is evident. Figure S2. Fluorescence image of nuclei suspensions prepared with Sorbitol-based buffer. Nuclei were generally solitary without evident clumping.
