# Supplementary material for: A Novel Sorbitol-Based Flow Cytometry Buffer Is Effective for Genome Size Estimation across a Cypriot Grapevine Collection
Source: Plants (Basel). 2024 Mar 5;13(5):733. doi: 10.3390/plants13050733 (PMC10933969; doi:10.3390/plants13050733)
Supplement: Supplementary file 1 [file plants-13-00733-s001.zip › plants-2864281-supplementary.pdf]

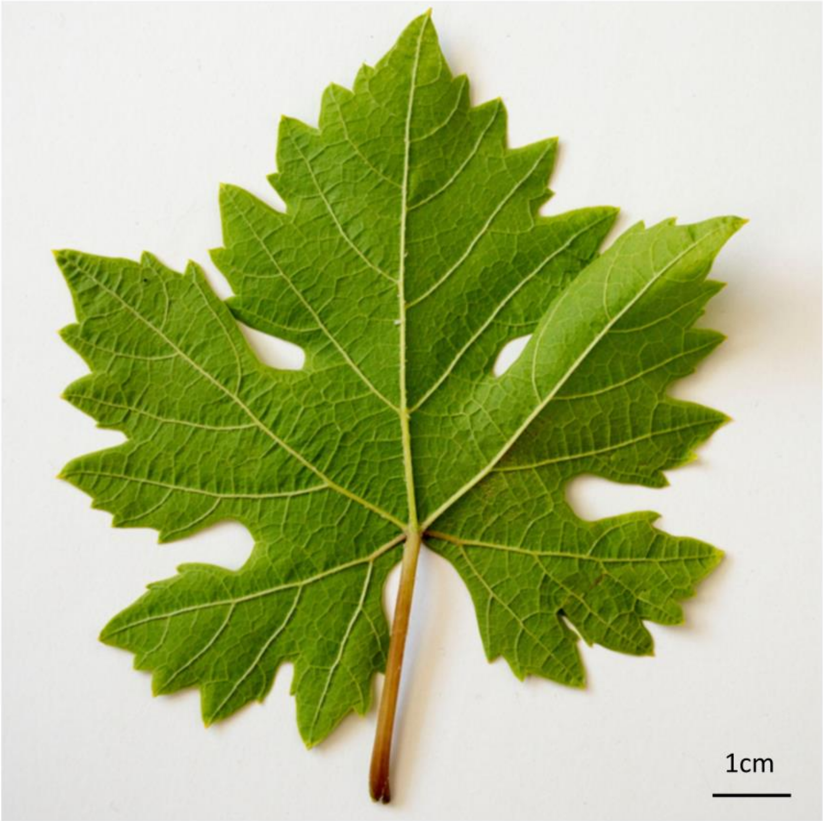

Figure S1. An example of a fully matured leaf of cv. 'Xynisteri'. Accumulation of pigments due to UV exposure is evident.

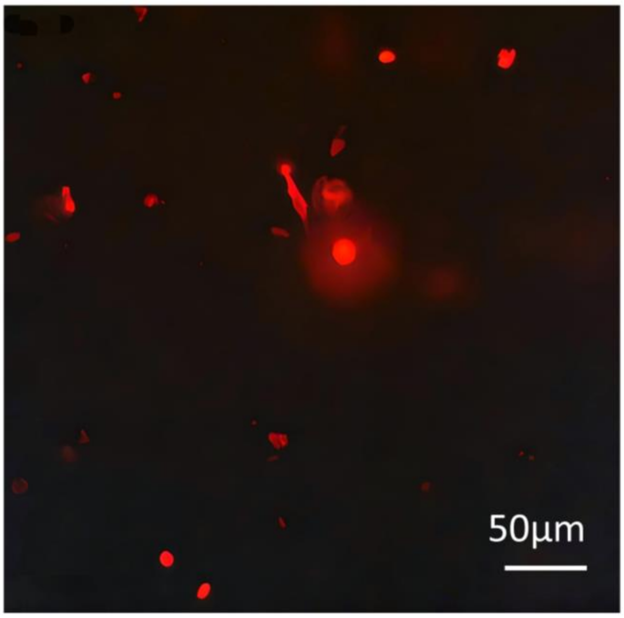

Figure S2. Fluorescence image of nuclei suspensions prepared with Sorbitol-based buffer. Nuclei were generally solitary without evident clumping.
